# Dynamical and thermodynamical approaches to open quantum systems

**DOI:** 10.1038/s41598-020-59241-7

**Published:** 2020-02-13

**Authors:** Vitalii Semin, Francesco Petruccione

**Affiliations:** 1 Samara National Research University, Samara, 443081 Russia; 2 Quantum Research Group, School of Chemistry and Physics, University of KwaZulu-Natal, Durban, 4001 South Africa; 3 National Institute for Theoretical Physics (NITheP), KwaZulu-Natal, South Africa; 4 School of Electrical Engineering, KAIST, Daejeon, 34141 Republic of Korea

**Keywords:** Quantum mechanics, Qubits

## Abstract

The non-Markovian dynamics of open quantum systems is studied from two different points of view. The first one coincides with the traditional tracing out of the environmental degrees of freedom, presented in classical textbooks on open quantum systems. The second one is an approximation of the exact density operator with the knowledge of only several dynamical variables in the spirit of non-equilibrium thermodynamics. The approximation is based on the principle of maximal entropy. We discuss the information and the Renyi entropies, which lead to different approximations. The time-convolutionless master equation governs the dynamics of both traditional and approximated reduced density operator with a particular projection operator. Considering the example of two interacting qubits in a thermal environment, we compare the traditional and thermodynamical approaches.

## Introduction

The theory of open quantum systems is an extremely multilateral field of research and operates with many different methods and ideas behind it. From the formally exact Hu-Paz-Zhang master equation^1^ for a harmonic oscillator in a bath of harmonic oscillators to the axiomatic Lindblad-Gorini-Kossakowski-Sudarshan master equation^2,3^, from the Nakajima-Zwanzig generalised integrodifferential master equation^4,5^ to the time-convolutionless (TCL) generalised master equation^6^, from the phenomenological post-Markovian master equation^7^ to the Stochastic Schrödinger equation^8^ this is a small part of methods existing in the theory of open quantum systems. Many of these methods are applicable in quantum optics^9^, quantum computations^10,11^, condensed matter physics^12,13^, and the theory of decoherence^14^.

The modern experimental setup allows studying the ultra-short dynamics of quantum systems for time scales much shorter than the characteristic relaxation times of quantum systems. At such time scales, the quantum systems show non-Markovian dynamics essentially^6^. The traditional theory of open quantum systems^6^ gives powerful methods to study low-dimensional open quantum systems in non-Markovian regimes. Unfortunately, these methods are hardly applicable to describe high-dimensional systems, because they lead to large systems of differential equations. In this article, we study the possible strategies to overcome this difficulty.

We suggest an approach based on the principle of maximal entropy, which is similar to the non-equilibrium thermodynamical methods. Each particular form of entropy, such as the information or Renyi ones, leads to some form of the reduced density operator, which can be used to approximate the exact reduced density operator with the knowledge of only several degrees of freedom of the system. The universal time-convolutionless master equation governs the dynamics of the approximated reduced density operator with a particular type of projection operator. We examine the introduced thermodynamical ideas on the example of two interacting qubits in a common environment and compare the results given by different approximations with the traditional one. We show that the traditional reduced density operator of qubits may be successfully approximated with the knowledge of only four degrees of freedom.

The article is organised as follows. In Sec. II we derive the general form of universal local in time master equation for an arbitrary projection operator. Sec. III deals with the main ideas of the theory of open quantum systems and ways to describe them. In Sec. IV we show for the particular example of two interacting qubits in a common environment the workability of the considered methods. Finally, in Sec. V, we discuss some alternative strategies to approximate the reduced density operator of open quantum systems and conclude.

## Time-Convolutionless Master Equation

The main object of this paper is a projection operator. Let ${\mathscr{P}}$ be a projection operator which projects a state of a whole studied system onto some relevant subspace and ${\mathscr{Q}}=1-{\mathscr{P}}$ be the complementary projection operator. In this section we are not interested in the exact form of these operators, but we require their main properties:

1 $$\begin{array}{rcl}{{\mathscr{P}}}^{2} & = & {\mathscr{P}},\\ {{\mathscr{Q}}}^{2} & = & {\mathscr{Q}},\\ {\mathscr{P}}{\mathscr{Q}} & = & {\mathscr{Q}}{\mathscr{P}}=0.\end{array}$$

Assume also that the projection operators commute with the time-derivative ${\mathscr{P}}\frac{\partial }{\partial t}=\frac{\partial }{\partial t}{\mathscr{P}}$ and ${\mathscr{Q}}\frac{\partial }{\partial t}=\frac{\partial }{\partial t}{\mathscr{Q}}.$ Now, we are ready to derive the general form of the time-convolutionless (TCL) master equation.

By acting the projection operators onto both sides of the Liouville equation we obtain

2 $$\frac{\partial }{\partial t}{\mathscr{P}}\rho ={\mathscr{P}}{\mathscr{L}}{\mathscr{P}}\rho (t)+{\mathscr{P}}{\mathscr{L}}{\mathscr{Q}}\rho (t),$$

3 $$\frac{\partial }{\partial t}{\mathscr{Q}}\rho ={\mathscr{P}}{\mathscr{L}}{\mathscr{P}}\rho (t)+{\mathscr{Q}}{\mathscr{L}}{\mathscr{P}}\rho (t),$$

where ${\mathscr{L}}{A}=-\,{i}[{H},{A}]$ is the Liouville superoperator, *H* is the system Hamiltonian, and *ρ* is the density operator. The formal solution of Eq. (3) reads

4 $${\mathscr{Q}}\rho (t)={\mathscr{G}}(t,{t}_{0}){\mathscr{Q}}\rho ({t}_{0})+{\int }_{{t}_{0}}^{t}ds{\mathscr{G}}(t,s){\mathscr{Q}}{\mathscr{L}}{\mathscr{P}}\rho (s),$$

where we introduce the propagator ${\mathscr{G}}(t,{t}_{0})=ex{p}_{-}[{\int }_{{t}_{0}}^{t}{\mathscr{Q}}{\mathscr{L}}ds]$ and exp_±_ is the (anti)chronological exponent. The solution of Eq. (4) together with Eq. (2) leads to the famous Nakajima-Zwanzig integro-differential master equation. This master equation is not convenient for us, since in particular cases, it may be non linear, as well as being difficult to solve due to its integro-differential form. To overcome this difficulty we substitute the identity $\rho (s)=G(t,s)({\mathscr{P}}+{\mathscr{Q}})\rho (t),$ where $G(t,s)={\exp }_{+}[-\,{\int }_{s}^{t}{\mathscr{L}}ds],$ into the right hand side of Eq. (4) and, after some algebra, we come to

5 $${\mathscr{Q}}\rho (t)={\mathrm{(1}-\Theta (t))}^{-1}\Theta {\mathscr{P}}\rho (t)+{\mathrm{(1}-\Theta (t))}^{-1}{\mathscr{G}}(t,{t}_{0}){\mathscr{Q}}\rho ({t}_{0}),$$

where $$\Theta (t)={\int }_{{t}_{0}}^{t}\,{ds}{\mathscr{G}}(t,s){\mathscr{Q}}{\mathscr{L}}{\mathscr{P}}G(t,s\mathrm{)}.$$

Substituting Eq. (5) into Eq. (2) then gives the general form of the TCL master equation

6 $$\frac{\partial }{\partial t}{\mathscr{P}}\rho ={\mathscr{K}}(t){\mathscr{P}}\rho (t)+{\mathscr{T}}(t){\mathscr{Q}}\rho ({t}_{0}),$$

with $${\mathscr{K}}(t)={\mathscr{P}}{\mathscr{L}}{\mathrm{(1}-\Theta (t))}^{-1}{\mathscr{P}}$$ and $${\mathscr{L}}(t)={\mathscr{P}}{\mathscr{L}}{\mathrm{(1}-\Theta (t))}^{-1}{\mathscr{G}}(t,{t}_{0}){\mathscr{Q}}.$$

The above TCL master equation is usually studied perturbatively and up to the second order expansion the generators are

7 $${\mathscr{K}}(t)={\mathscr{P}}{\mathscr{L}}(t)\{\mathrm{[1}+{\int }_{{t}_{0}}^{t}\,ds{\mathscr{Q}}{\mathscr{L}}(s)]{\mathscr{P}}\},$$

8 $${\mathscr{I}}(t)={\mathscr{P}}{\mathscr{L}}(t)\{[1+{\int }_{{t}_{0}}^{t}ds{\mathscr{Q}}{\mathscr{L}}(s)]{\mathscr{Q}}\}.$$

Equation (6) together with the generators (7)–(8) is the main master equation, which arises in many applications, for example, the famous Lindblad-Gorini-Kossakowski-Sudarshan Markovian master equation^2,3^ is a particular case of Eq. (6). Usually, the inhomogeneity ${\mathscr{I}}(t)$ is put equal to zero by a particular choice of the initial conditions, but this is not possible in general, and we explicitly keep this term. Notice that the locality in time of the left-hand side of the master equation allows implementing an efficient numerical algorithm to study the dynamics of the system even in non-Markovian regimes.

The general form of Eq. (6) encodes an infinite number of master equations for an infinite number of different projection operators. All the master equations are equivalent in some sense and reproduce the exact dynamics with the same precision in general, but a fortuned choice of the relevant subspace may lead to better results. Below we describe possible forms of projection operators and ideas lying behind them for describing open quantum systems.

## Description of Open Quantum Systems

Traditionally, an open quantum system is understood as a system interacting with its environment. An open system is characterised by this interaction and properties of the environment: it is clear that an atom in the vacuum is not equivalent to the same atom in a crystal. Very often the interaction between an open system and the environment is assumed to be weak. This assumption allows one to successfully study an open system using the TCL master equation with the generators (7–8).

The environment usually has many–often infinitely many–degrees of freedom and is characterised by its energy spectrum. The spectrum may be both discrete and continuous, with energy gaps or not. Of course, each concrete case needs to be considered individually in the context of the specific problem. Nevertheless, we can indicate general assumptions, which simplify the consideration of an open system and are used quite often. First of all, the system and its environment are assumed to be uncorrelated at the initial moment of time. Secondly, the initial environment state is usually fixed and corresponds to some stable equilibrium, thermal or squeezed. Thirdly, the environment is modeled as a very inert system, and its state does not change significantly during the evolution of the open system, and such changes of state are neglected. The last is usually referred as the Born approximation.

The above three assumptions give some general ideas about the environment. Now, we are interested in the evolution of an open system itself. Here we can indicate two different ways to deal with an open system, which we can call dynamical and thermodynamical.

### Dynamical approach

The dynamical method is a basis of the traditional theory of open quantum systems. The main feature of this method is its relative simplicity. Here, one does not assume any specific structure of the density operator of an open quantum system, but instead considers it as a matrix, which may have an infinite number of dimensions. The aim is to describe the dynamics of some particular matrix elements of the density matrix. For example, for some reason, one is interested only diagonal elements of the density operator or some particular off-diagonal elements. The extraction of the necessary elements is convenient to perform with the help of projection operators.

The projection operator which extracts the necessary degrees of freedom and is consistent with the Born approximation has the following general structure

9 $${\mathscr{P}}\rho =\sum _{ij}\,{\rm{Tr}}({E}_{ij}\rho )\frac{{E}_{ij}^{\dagger }\otimes {\rho }_{E}}{{\rm{Tr}}{E}_{ij}^{\dagger }{E}_{ij}},{\rm{Tr}}{E}_{ij}{E}_{kl}^{\dagger }={\delta }_{ik}{\delta }_{jl},$$

where $\rho$ is the arbitrary density operator, ${\rho }_{E}$ is the density operator of a fixed state of environment, and ${\delta }_{ij}$ is the Kronecker delta symbol. In principle, matrices ${E}_{ij}$ may form any orthogonal set, but the most reasonable choice of ${E}_{ij}$ is the matrices with only one unit in the intersection of $i$th row and $j$th column and 0 elsewhere. A particular case of Eq. (9) is the projection operator

10 $${\mathscr{P}}\rho ={{\rm{Tr}}}_{E}(\rho )\otimes {\rho }_{E},$$

which is traditionally used in the theory of open quantum systems. In the above equation ${{\rm{Tr}}}_{E}$ denotes the partial trace over environmental degrees of freedom. Notice that the second order TCL master Eq. (6) with the projection operator (10) represents one of the most widely used types of a master equation.

Sometimes it is necessary to include into consideration also some changes in the environment, and for this reason, one may use the so-called correlated projection operators^15–20^. The correlated projection operators are also a part of the dynamical approach, but we do not consider them in this article.

It is worth noting that the dynamical approach with the projector (10) or correlated projectors is challenging to implement for describing high-dimensional open quantum systems, since the TCL master equation represents a system of a vast number of equations, which may, for example, appear in condensed matter physics. Below we show the way to overcome this difficulty.

### Thermodynamical approach

Another way to investigate an open quantum system comes from non-equilibrium thermodynamics. Conventional problems of thermodynamics consist of billions of degrees of freedom, and the solution of the exact Liouville equation is not possible. Thermodynamic approaches overcome this problem by assuming that the system could be approximated with the knowledge of a few relevant degrees of freedom of the system. The larger the number of variables that are taken into account, then the better the approximation one builds. For instance, the well known Gibbs ensemble approximates the real equilibrium state with the help of only several additive integrals of motion, such as energy, momentum and angular momentum. Moving further from the equilibrium the Gibbs ensemble gives a purer approximation of the real state of the system.

The first question is how to choose the necessary set of relevant degrees of freedom. According to the modern non-equilibrium thermodynamics^21^ the evolution of the system goes through several consecutive stages. The defined set of variables characterises each stage, and the number of these variables is fixed. If we assume the stage of evolution, we can choose the set. Generally, the most reasonable choice is ${a}_{i}^{\dagger }{a}_{k},$ for all possible combinations of the creation (annihilation) operators of components of the quantum system. This choice is a compromise between difficulty of the description and accuracy of approximation. Of course, the set of relevant variables depends on a concrete problem and may vary, but in general the above set is enough.

The next question is an approximation of the real density operator of the system, i.e. a solution of the Liouville equation, through the chosen set of variables. First of all, we assume that we know a functional form of the density operator. Let this form be

11 $${\rho }_{r}=F({P}_{m},t),$$

where ${P}_{m}$ are the chosen relevant variables. We assume that this functional form is preserved during the evolution. Of course, we want that the real averages of the variables coincide with the averaging for ${\rho }_{r},$ i.e.,

12 $${\langle {P}_{m}\rangle }^{t}={\rm{Tr}}({P}_{m}{\rho }_{r}),$$

where ${\langle {P}_{m}\rangle }^{t}$ is the real expectation value of the variables, and the superscript indicates the time-dependence. The expressions (12) are called the self-consistency conditions. The functional (11), satisfying the self-consistency conditions, is referred to as a reduced density operator. The conditions (12) are actually a system of non-linear equation that gives connection between the dynamical variables and thermodynamical parameters.

Further, we have to find the evolution of the reduced density operator (11). For this reason we introduce the Kawasaki-Gunton projection operator with the property ${\mathscr{P}}\rho ={\rho }_{r},$ which has the following explicit form^22^

13 $${\mathscr{P}}(t)A={\rho }_{{\rm{r}}}(t){\rm{Tr}}A+\sum _{m}\,\,\{{\rm{Tr}}(A{P}_{m})-({\rm{Tr}}A){\langle {P}_{m}\rangle }^{t}\}\frac{\partial {\rho }_{{\rm{r}}}(t)}{\partial {\langle {P}_{m}\rangle }^{t}}.$$

The TCL master Eq. (6) with the Kawasaki-Gunton projection operator (13) defines the dynamics of the reduced density operator (11).

Now we must clarify the concrete functional form of the reduced density operator. Familiar reduced density operators follow from the principle of maximal entropy. There are several different forms of entropies, such as information, Renyi, Tsallis and others which can be used to build several types of reduced density operators.

In this paper we focus on two density operators, namely, the Gibbs-like density operator^21^

14 $${\rho }_{G}=\exp [\,-\,\sum _{m}\,{F}_{m}(t){P}_{m}]/{\rm{Trexp}}[\,-\,\sum _{m}\,{F}_{m}(t){P}_{m}],$$

which maximizes the information entropy ${S}_{I}={\rm{Tr}}\rho ln\rho ,$ and also the density operator which maximizes the Renyi entropy ${S}_{R}=\mathrm{1/(1}-q)\,{\rm{lnTr}}{\rho }^{q}$ ^23^,

15 $${\rho }_{R}=\frac{1}{{Z}_{R}}{(1+\frac{q-1}{q}\sum _{m}{F}_{m}({\langle {P}_{m}\rangle }^{t}-{P}_{m}))}^{\frac{1}{q-1}},$$

where ${Z}_{R}={\rm{Tr}}{(1+\frac{q-1}{q}{\sum }_{m}{F}_{m}({\langle {P}_{m}\rangle }^{t}-{P}_{m}))}^{\frac{1}{q-1}}$ normalizes. The multipliers ${F}_{m}$ define by the self-consistency conditions (12) and $q\ne 1$ is a free parameter.

It is interesting to note that ${lim}_{q\to 1}{\rho }_{R}={\rho }_{G}.$ This is just a consequence of the known limit for scalar functions of $x,$ namely, ${lim}_{q\to 1}{(1+\frac{q-1}{q}x)}^{\frac{1}{q-1}}=\exp (x\mathrm{)}.$ By using the definition of a matrix function $f(M)=U{\rm{diag}}\{f({\lambda }_{1}),\,\ldots ,$ $f({\lambda }_{m})\}{U}^{-1},$ where $U$ is the matrix consisting of eigenvectors and ${\lambda }_{k}$ is eigevalues of $M$, one can easily generalized the scalar identity to the matrix case. As result we have

16 $$\mathop{\mathrm{lim}}\limits_{q\to 1}{\rho }_{R}=\exp (\sum _{m}\,{F}_{m}({\langle {P}_{m}\rangle }^{t}-{P}_{m}))/{\rm{Tr}}\,\exp (\sum _{m}\,{F}_{m}({\langle {P}_{m}\rangle }^{t}-{P}_{m}))$$

$$=\exp (\sum _{m}\,{F}_{m}{\langle {P}_{m}\rangle }^{t})\exp (-\sum _{m}\,{F}_{m}{P}_{m})/(\exp (\sum _{m}\,{F}_{m}{\langle {P}_{m}\rangle }^{t}){\rm{Tr}}\,\exp (-\sum _{m}\,{F}_{m}{P}_{m}))={\rho }_{G}.$$

To effectively apply thermodynamical ideas to open quantum systems we assume that the system-environment interaction is weak, and that the reduced density operator has the following general form consistences with the previous assumptions mentioned about the bath

17 $${\rho }_{r}={\rho }_{i}\otimes {\rho }_{E}.$$

Here, the environment density operator ${\rho }_{E}$ does not depend on time and represent some equilibrium state of the bath. The system density operator ${\rho }_{i}$ has one of the forms of either (14) or (15) and depends only on system variables.

Notice that if one chooses all possible dynamical variables, i.e. all elements of the density operator as being relevant, one equivalently may use the projection operator (10) and derive the identical results without solution of the self-consistency condition (12). For this reason the thermodynamical approach is more flexible since one may construct any functional form of the reduced density operator. The parameters ${F}_{m}(t)$ can be considered as the non-equilibrium thermodynamical parameters, as for the operator (14) they coincide with the traditional definition of temperature, pressure and so on, while for (15) they do not have such a clear physical meaning^24^.

Below we show an example using the above ideas for the description of an open quantum system.

### Example. Two interaction qubits in a thermal environment

In this section, we compare the different approaches discussed above on the example of two interacting qubits in a thermal environment. We choose this example as the simplest model for which the traditional form of TCL master equation is already quite difficult to study. The model Hamiltonian is

18 $$\begin{array}{rcl}H & = & {\omega }_{0}\mathop{\sum }\limits_{i\mathrm{=1}}^{2}\,{\sigma }_{z}^{i}+\Omega ({\sigma }_{+}^{1}{\sigma }_{-}^{2}+{\sigma }_{+}^{2}{\sigma }_{-}^{1})+\sum _{j}\,{\omega }_{j}{b}_{j}^{\dagger }{b}_{j}\\  &  & +\,\sum _{i}\,\sum _{k}\,{g}_{k}({\sigma }_{-}^{i}{b}_{k}^{\dagger }{e}^{-i\overrightarrow{k}{\overrightarrow{r}}_{i}}+{\rm{h}}{\rm{.c}}.\,),\end{array}$$

where ${\sigma }_{i}^{j}$ are the Pauli matrices of $j$th qubit, ${\omega }_{0}$ is the qubit transition frequency, Ω is the constant of dipole-dipole interaction, ${b}_{j}^{\dagger }$ and ${b}_{j}$ are creation and annihilation operators of $j$th photon in the thermostat with frequency ${\omega }_{j},$ ${g}_{k}$ is the constant of atom-environment interaction, $\overrightarrow{k}$ is the qubit wave vector and ${\overrightarrow{r}}_{i}$ is the radius-vector of $i$-th qubit.

For convenience we transform the Hamiltonian into the interaction picture with respect to the Hamiltonian ${H}_{0}={\omega }_{0}{\sum }_{i\mathrm{=1}}^{2}\,{\sigma }_{z}^{i}+\Omega ({\sigma }_{+}^{1}{\sigma }_{-}^{2}+{\sigma }_{+}^{2}{\sigma }_{-}^{1})+{\sum }_{j}\,{\omega }_{j}{b}_{j}^{\dagger }{b}_{j}.$ The result is

19 $$V=\sum _{i}\,\sum _{k}\,{g}_{k}({\tilde{\sigma }}_{-}^{i}(t){b}_{k}^{\dagger }{e}^{-i\overrightarrow{k}{\overrightarrow{r}}_{i}+i{\omega }_{k}t}+{\rm{h}}{\rm{.c}}.\,),$$

where $${\tilde{\sigma }}_{-}^{i}(t)=\exp \,[i{H}_{0}t]{\sigma }_{-}^{i}\,\exp [\,-\,i{H}_{0}t\mathrm{]}.$$

We want to compare the three types of reduced density operators introduced above. The dynamical reduced density operator (10) has the form

20 $${\rho }_{S}={{\rm{Tr}}}_{E}(\rho )\otimes {\rho }_{E},$$

where $\rho$ is the solution of Liouville equation. To use the thermodynamical ideas, we choose the set of four relevant variables ${\sigma }_{z}^{1},{\sigma }_{z}^{2},{\sigma }_{+}^{2}{\sigma }_{-}^{1}$ and ${\sigma }_{+}^{1}{\sigma }_{-}^{2},$ which is consistent with the non-equilibrium thermodynamical recommendations^21,25^ and indicated above. The Gibbs-like density operator (14) is

21 $${\rho }_{G}=\frac{\exp [\,-\,\sum _{i}\,{\beta }_{i}(t){\sigma }_{z}^{i}-{f}_{1}(t){\sigma }_{+}^{1}{\sigma }_{-}^{2}-{f}_{2}(t){\sigma }_{+}^{2}{\sigma }_{-}^{1}]}{{\rm{Tr}}\,\exp [\,\,-\,\sum _{i}\,{\beta }_{i}(t){\sigma }_{z}^{i}-{f}_{1}(t){\sigma }_{+}^{1}{\sigma }_{-}^{2}-{f}_{2}(t){\sigma }_{+}^{2}{\sigma }_{-}^{1}]}\otimes {\rho }_{E},$$

and the Renyi density operator with parameter $q=2$ is

22 $$\begin{array}{rcl}{\rho }_{R} & = & \frac{1}{{Z}_{R}}(1+\frac{1}{2}\sum _{i}\,{B}_{i}(t)({\langle {\sigma }_{z}^{i}\rangle }^{t}-{\sigma }_{z}^{i})\\  &  & +\,\frac{1}{2}{F}_{1}(t)({\langle {\sigma }_{+}^{1}{\sigma }_{-}^{2}\rangle }^{t}-{\sigma }_{+}^{1}{\sigma }_{-}^{2})\\  &  & +\,\frac{1}{2}{F}_{2}(t)({\langle {\sigma }_{+}^{2}{\sigma }_{-}^{1}\rangle }^{t}-{\sigma }_{+}^{2}{\sigma }_{-}^{1}))\otimes {\rho }_{E},\,\end{array}$$

where ${\rho }_{E}=\exp [\,-\,\beta {\sum }_{j}\,{\omega }_{j}{b}_{j}^{\dagger }{b}_{j}]/{\rm{Tr}}\,\exp [\,-\,\beta {\sum }_{j}\,{\omega }_{j}{b}_{j}^{\dagger }{b}_{j}]$ is the equilibrium density operator of the bath with the inverse temperature $\beta$.

We use the parameter $q=2$ in Eq. (22) to simplify the consideration. Of course, the results of the approach will depend significantly on the specific choice of this parameter and require some additional study. We plan to consider the most appropriate value of the parameter in our future works.

Notice that the dimension of the open quantum system Hilbert space is 4, such that the reduced density operator has dimension 16. We try to approximate the reduced density operator by (21)–(22) with the help of only four parameters. It is not difficult to understand that the relevant parameters, in this case, are just parts of the system Hamiltonian.

We choose the initial condition for the open system to be

23 $$\rho \mathrm{(0)}=\mathrm{1/4}{{\bf{I}}}_{4\times 4}\otimes {\rho }_{E}.$$

In this case the inhomogeneity in Eq. (6) cancels for all three reduced density operators (20)–(22), so that the second order TCL master equation has the form

24 $$\frac{\partial {\rho }_{i}}{\partial t}=-\,{\int }_{0}^{t}\,{\mathscr{P}}[V(t),[V(s),{\rho }_{i}(t)]ds,$$

where ${\rho }_{i}$ is one of (20)–(22) and ${\mathscr{P}}$ is corresponding projection operator (10) or (13). To derive (24) we used the fact that for the considered model ${\mathscr{P}} {\mathcal L} {\mathscr{P}}=0$ for the both projection operators (10) and (13). More explicitly, the master equation reads

25 $$\begin{array}{rcl}{\dot{\rho }}_{i}(t) & = & {\int }_{0}^{t}d{t}_{1}{\mathscr{P}}\{[{K}^{\dagger }({t}_{1}){\rho }_{i}(t)K(t)-K(t){K}^{\dagger }({t}_{1}){\rho }_{i}(t)]L\\  &  & +\,\,\,\,\,\,\,\,\,\,\,\,\,\,\,[K({t}_{1}){\rho }_{i}(t){K}^{\dagger }(t)-{K}^{\dagger }(t)K({t}_{1}){\rho }_{i}(t)]N+{\rm{h}}{\rm{.c}}\},\,\,\,\,\,\,\,\end{array}$$

where $L={\int }_{0}^{\infty }\,d\omega J(\omega )(\coth (\beta \omega \mathrm{/2)}+\mathrm{1)/2}{e}^{i({\omega }_{0}-\omega )(t-{t}_{1})}$ and $N={\int }_{0}^{\infty }\,d\omega J(\omega )(\coth (\beta \omega \mathrm{/2)}-\mathrm{1)/2}{e}^{-i({\omega }_{0}-\omega )(t-{t}_{1})},$ and $J(\omega )$ is the bath spectral density, $K(t)=({P}_{+}^{1}R({\alpha }_{2},\,-\,{\alpha }_{1})+{P}_{-}^{1}R({\alpha }_{2},{\alpha }_{1})){\sigma }_{+}^{2}+\mathrm{(1}\leftrightarrow \mathrm{2)}$, ${P}_{+}^{i}={\sigma }_{+}^{i}{\sigma }_{-}^{i},$ ${P}_{-}^{i}={\sigma }_{-}^{i}{\sigma }_{+}^{i},$ and $R(\alpha ,\beta )=\alpha \,\cos (t\Omega )+i\beta \,\sin (t\Omega ),$ and ${\alpha }_{j}={e}^{i{\overrightarrow{k}}_{j}{\overrightarrow{r}}_{j}}.$

By choosing the spectral density of the bath to take the form $J(\omega )=\lambda \omega \,\exp [\,-\,\omega /W],$ where $W$ is the cut-off frequency and $\lambda$ is actually the constant of interaction between the qubit and the bath, we can numerically solve Eq. (25), although each density operator (20), (21) and (22) should considered separately. The form of the spectral density is not crucial for our analysis and, in principle, can be replaced by any other form. The chosen form is dictated by the ability to analytically take integrals over the frequency domain.

### Dynamical approach

The master Eq. (25) with the projection operator (10) represents the classical result of the theory of open quantum systems for a non-Markovian case. Here the master equation represents a system of 16 linear differential equations. The analytical solution of this system is not possible to derive due to non-trivial time-dependence of the coefficients, which obeys a double integration over both frequency and time. Since, the integration can be calculated analytically only over one of these domains, therefore, the numerical integration of this equation is highly problematic in the non-Markovian case.

To calculate the average value of some quantity $A$ one must transform the solution of the master Eq. (25) back to the Schrödinger picture, namely

26 $$\langle A\rangle ={\rm{Tr}}(\exp (i{H}_{0}t){\rho }_{i}\,\exp (\,-\,i{H}_{0}t)A),$$

where ${\rho }_{i}$ is one of the introduced reduced density operators.

**Figure 1:**
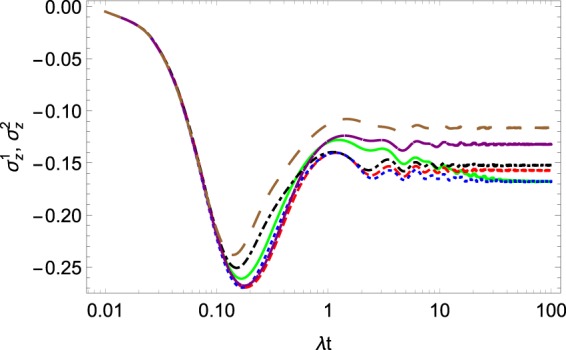
The evolution of $$\langle {\sigma }_{z}^{1}\rangle $$ and $$\langle {\sigma }_{z}^{2}\rangle $$. The green solid is $$\langle {\sigma }_{z}^{2}\rangle $$ and the blue dotted is $$\langle {\sigma }_{z}^{1}\rangle ,$$ following from the dynamical approach, the purple dashed is $$\langle {\sigma }_{z}^{2}\rangle $$ and the red dashed is $$\langle {\sigma }_{z}^{1}\rangle ,$$ following from the Gibbs-like thermodynamical approach, the brown long-dashed is $$\langle {\sigma }_{z}^{2}\rangle $$ and the black dot-dashed is $$\langle {\sigma }_{z}^{1}\rangle ,$$ following from the Renyi thermodynamical approach. The system parameters are $$\Omega =0.6\lambda ,W=10\lambda ,{\omega }_{0}=2\lambda ,\beta =0.3,$$$$\,{\alpha }_{k}=\exp [\pi ik/4]$$.

The results of the dynamical approach for the dynamics of $\langle {\sigma }_{z}^{1}\rangle$ and $\langle {\sigma }_{z}^{2}\rangle$ are presented in Fig. 1. One can see that the both curves came to the same equilibrium states and only differs at initial time. The last is due to differences in position factor ${\alpha }_{k}$.

**Figure 2:**
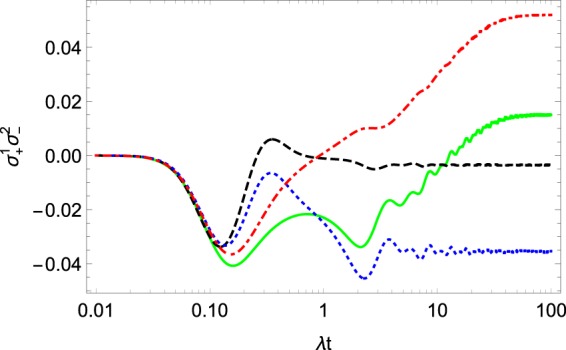
The evolution of $\langle {\sigma }_{+}^{1}{\sigma }_{-}^{2}\rangle .$ The green solid and red dot-dashed curves are $\Re \langle {\sigma }_{+}^{1}{\sigma }_{-}^{2}\rangle$ and $\Im \langle {\sigma }_{+}^{1}{\sigma }_{-}^{2}\rangle ,$ respectively, for density operator (20), and the blue dotted and black dashed curves are $\Re \langle {\sigma }_{+}^{1}{\sigma }_{-}^{2}\rangle$ and $\Im \langle {\sigma }_{+}^{1}{\sigma }_{-}^{2}\rangle$ for the density operator (29). The system parameters are $\Omega =0.6\lambda ,W=10\lambda ,{\omega }_{0}=2\lambda ,\beta =0.3,{\alpha }_{k}=\exp [\pi ik\mathrm{/4]}$.

**Figure 3:**
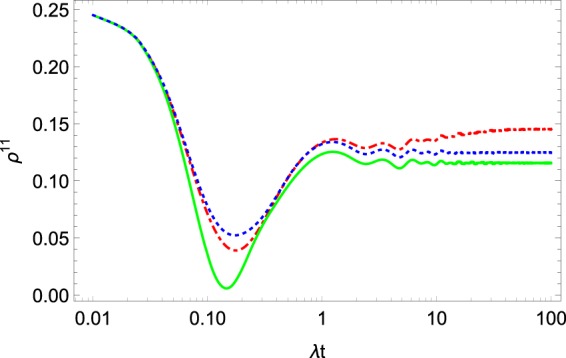
Collective exited state ${\rho }_{R}^{11}$ (green solid curve), ${\rho }_{G}^{11}$ (blue dotted curve) and ${\rho }_{S}^{11}$ (red dot-dashed curve). The system parameters are $\Omega =0.6\lambda ,W=10\lambda ,{\omega }_{0}=2\lambda ,\beta =\mathrm{0.3,}\,{\alpha }_{k}=\exp [\pi ik\mathrm{/4]}$.

The dynamics of $\langle {\sigma }_{+}^{1}{\sigma }_{-}^{2}\rangle$ and ${\rho }^{11}$ can be seen in Figs. 2 and 3. All the observables come to some equilibrium value. This fact is in agreement with physical intuition. Remarkably, the dynamics describes the initial oscillations of the observables. Such oscillations often appear in the non-Markovian description of open quantum systems^6,25^ and may be considered as a specific property of non-Markovian systems^25^.

### Gibbs-like density operator

To handle the different variants of thermodynamical approach one needs to specify the action of the Kawasaki-Gunton projection operator (13). Clearly, since ${\rm{Tr}}( {\mathcal L} A)=\mathrm{0,}$ the Kawasaki-Gunton projection operator on the right-hand side of Eq. (25) can be replaced by the simpler Robertson projection operator

27 $${\mathscr{P}}(t)A=\sum _{m}\,\,\{{\rm{Tr}}(A{P}_{m})\}\frac{\partial {\rho }_{{\rm{R}}}(t)}{\partial {\langle {P}_{m}\rangle }^{t}}.$$

By then multiplying both sides of the master Eq. (25) on some relevant variables ${P}_{k},$ and taking the trace, one arrives at the transport equation.

28 $$\begin{array}{rcl}{\langle {\dot{P}}_{k}\rangle }^{t} & = & {\int }_{0}^{t}d{t}_{1}{\rm{Tr}}{P}_{k}\{[{K}^{\dagger }({t}_{1}){\rho }_{i}(t)K(t)-K(t){K}^{\dagger }({t}_{1}){\rho }_{i}(t)]L\\  &  & +[K({t}_{1}){\rho }_{i}(t){K}^{\dagger }(t)-{K}^{\dagger }(t)K({t}_{1}){\rho }_{i}(t)]N+{\rm{h}}{\rm{.c}}\}.\end{array}$$

Repeating this procedure for all relevant variables allows one to derive the system of transport equations, the solution of which completely determines the time evolution of the reduced density operator. Notice that the system of the transport equations is completely equivalent to (25). The most difficult part here is the calculation of the trace on the right-hand side of Eq. (28). To do this, one must express the reduced density operator in terms of the relevant variables, or in other words, one must solve the self-consistency conditions (12). These conditions usually represent a system of non-linear equations, which may not have analytical solution in general. Nevertheless, restricting number of relevant variables allows us to overcome such difficulties.

For the considered example the self-consistency conditions can be solved such that the reduced density operator has the following form in terms of relevant variables,

29 $${\rho }_{G}(t)=(\begin{array}{cccc}F & 0 & 0 & 0\\ 0 & \frac{1}{2}+\langle {\sigma }_{z}^{1}\rangle -F & \langle {\sigma }_{-}^{1}{\sigma }_{+}^{2}\rangle  & 0\\ 0 & \langle {\sigma }_{+}^{1}{\sigma }_{-}^{2}\rangle  & \frac{1}{2}+\langle {\sigma }_{z}^{2}\rangle -F & 0\\ 0 & 0 & 0 & {e}^{x}F\end{array}),$$

where $$F=\frac{\langle {\sigma }_{z}^{1}\rangle +\langle {\sigma }_{z}^{2}\rangle }{1-{e}^{x}}$$ and $$x=\,\log \,\frac{4\langle {\sigma }_{+}^{1}{\sigma }_{-}^{2}\rangle \langle {\sigma }_{+}^{2}{\sigma }_{-}^{1}\rangle -(2\langle {\sigma }_{z}^{1}\rangle -1)(2\langle {\sigma }_{z}^{2}\rangle -1)}{4\langle {\sigma }_{+}^{1}{\sigma }_{-}^{2}\rangle \langle {\sigma }_{+}^{2}{\sigma }_{-}^{1}\rangle -(2\langle {\sigma }_{z}^{1}\rangle +1)(2\langle {\sigma }_{z}^{2}\rangle +1)}.$$

With the known explicit form (29) the calculation of the right side of the transfer Eq. (28) is performed by multiplying matrices and presents no difficulties.

It is interesting that the matrix elements have a non-linear dependency on the relevant variables and, thus, the transport equations are non-linear. It is worth also noting that the non-linearity of the equations follows from the solution of the self-consistency conditions, i.e. from the particular functional form of the reduced density operator, and is not a feature of the general Eq. (6).

To determine the reduced operator (29) one needs only four equations, which are fewer than the number obtained from the dynamical approach discussed above. The results for $\langle {\sigma }_{z}^{\mathrm{1,2}}\rangle$ and for the dynamics of the real part and imaginary of $\langle {\sigma }_{+}^{1}{\sigma }_{-}^{2}\rangle$ are plotted in Figs. 1 and 2. On the figures, we show the dynamics of the same parameters following from the dynamical approach. One can see that for short time scales both approaches give very similar results while at long times the results differ. Another distinction is that the approximation with the help of (29) reaches equilibrium faster than its dynamical counterpart.

**Figure 4:**
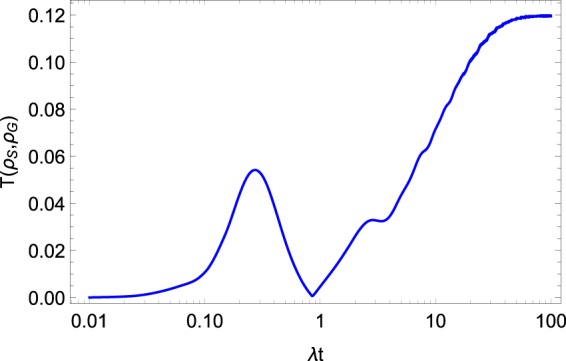
Trace distance $$T({\rho }_{S},{\rho }_{G}\mathrm{)}.$$ The system parameters are $$\Omega =0.6\lambda ,W=10\lambda ,{\omega }_{0}=2\lambda ,\beta =\mathrm{0.3,}$$$${\alpha }_{k}=\exp [\pi ik/\mathrm{4]}$$.

More accurate ideas about the difference between the dynamical and thermodynamical approaches discussed here are given in Fig. 4. In this figure we plot the trace distance between two reduced density operators, namely $T({\rho }_{S},{\rho }_{G})=1/2{\rm{Tr}}\sqrt{{({\rho }_{S}-{\rho }_{G})}^{2}}.$ It is clear that the trace distance for two density matrices may take any value between 0 and 1. So, the lesser this value the better approximation we have. One can see that the difference between the two density operators grows with time, but, in principle, does not exceed 12%, and the reduced operator (29) can be considered as a good alternative to ${\rho }_{S}.$

### Renyi density operator

As mentioned above, the transport equations for the considered model has the form (28) and are defined by the explicit form of the reduced density operator. The explicit form follows directly from the solution of the self-consistency conditions (12). The self-consistency conditions for the reduced operator (22) can than be solved, and the result for ${\rho }_{R}$ is

30 $${\rho }_{R}(t)=(\begin{array}{cccc}\frac{1+2{F}_{+}}{4} & 0 & 0 & 0\\ 0 & \frac{1+2{F}_{-}}{4} & \langle {\sigma }_{-}^{1}{\sigma }_{+}^{2}\rangle  & 0\\ 0 & \langle {\sigma }_{+}^{1}{\sigma }_{-}^{2}\rangle  & \frac{1-2{F}_{-}}{4} & 0\\ 0 & 0 & 0 & \frac{1-2{F}_{+}}{4}\end{array}),$$

where ${F}_{\pm }=\langle {\sigma }_{z}^{1}\rangle \pm \langle {\sigma }_{z}^{2}\rangle .$ One can see that for this specific case the matrix elements of ${\rho }_{R}$ depend linearly on the relevant variables, hence, the transport equations are linear.

**Figure 5:**
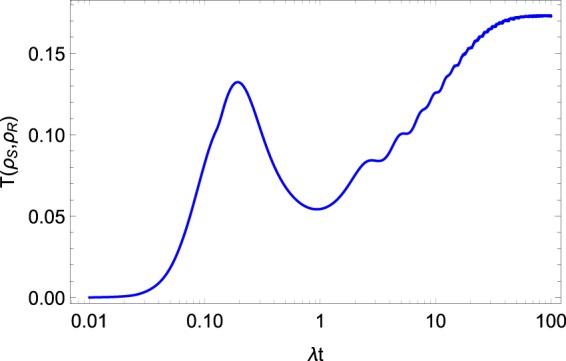
Trace distance $$T({\rho }_{S},{\rho }_{R}\mathrm{)}.$$ The system parameters $$\Omega =0.6\lambda ,W=10\lambda ,{\omega }_{0}=2\lambda ,\beta =0.3,$$$${\alpha }_{k}=\exp [\pi ik/4]$$.

The results of the solution of the master Eq. (25) for $\langle {\sigma }_{z}^{\mathrm{1,2}}\rangle$ and for the population of the collective exited state ${\rho }_{R}^{11}$ are presented in Figs. 1 and 3, respectively. On the same figures, we also plot the results following from above-discussed approaches. One can see that the results of all three approaches are similar, especially for the short-time dynamics. Nevertheless, note that the equilibrium value is distinguished for all the approaches. We plot the trace distance $T({\rho }_{S},{\rho }_{R})$ in Fig. 5. Here, one can see that the reduced density operator ${\rho }_{R}$ reproduces the density operator ${\rho }_{S}$ less accurately than the corresponding Gibbs-like operator ${\rho }_{G}.$ Nevertheless, the trace distance between the two operators ${\rho }_{S}$ and ${\rho }_{R}$ is much smaller than 1, so we can conclude that the operator ${\rho }_{R}$ also reproduces the result of the dynamical approach quite well, at least according to the trace distance criteria.

## Discussion and Outlook

In this paper, we considered two approaches for the description of open quantum systems. We indicated that along with the traditional approach, which we called the dynamical approach, there are a vast number of ways to approximate the density operator of an open quantum system, by a limited number of relevant degrees of freedom of the system. The second one we referred to as the thermodynamical approach. The main idea of the thermodynamical approach rotates around the possible functional form of the approximated density operator dependent on the relevant characteristics. Many functional forms of the approximated or reduced density operator follow from the principle of maximal entropy, and, of course, for each entropy one can derive a new functional form for the reduced density operator. We showed two possibilities for the information and Renyi entropies and, using the standard ideas about open quantum systems, applied these reduced density operators to describe open quantum systems.

As soon as the form of the reduced density operator is chosen the dynamics of this operator is governed by the TCL master equation with the appropriate projection operator. The equation may be non-linear, but this feature is a property of the chosen reduced density operator.

We demonstrated the application of both the dynamical and thermodynamical approaches to the system of two interacting qubits in a common environment. We showed that the traditional density operator could be successfully approximated by fewer parameters in the thermodynamical approach. We explicitly derived the expressions for the reduced density operators in the thermodynamical approach and indicated where the non-linearity of the TCL master equation appears. Using the trace distance as a measure of the accuracy of the approximation, we also showed that the thermodynamical approach can describe open quantum systems quite accurately.

The results of the Renyi thermodynamics is less accurate than the corresponding Gibbs-like thermodynamics. Nevertheless, both thermodynamical methods reproduced the qualitative behavior of the quantum dynamics of the considered system quite well. Both approaches reach equilibrium and show the initial oscillations of the parameters. Both approaches reproduce correctly the short-time dynamics. Nevertheless, the Renyi thermodynamics depends on the free parameter *q* and, probably, a better choice of this parameter will improve the results given by the approach.

The thermodynamical ideas arise in problems consisting of billions of degrees of freedom. This fact and the results of the present article derived for the simple model allows hoping that the suggested thermodynamical methods can be applied to models consisting of dozens of interacting particles. Such models are very interesting in the context of quantum informatics. The traditional dynamical approach fails because the dimensions of the problem is too large, while the thermodynamical ideas allows to reduce the number of relevant variables to the order of number of particle that can already be studied. In this respect the thermodynamical approach provides a novel perspective to the investigation of large quantum systems.
